# Medication adherence and associated factors among psychiatry patients at Asella Referral and Teaching Hospital in Oromia, Ethiopia: Institution based cross sectional study

**DOI:** 10.1371/journal.pone.0283829

**Published:** 2023-04-13

**Authors:** Dinkinesh Begna Gudeta, Kassech Leta, Birhanu Alemu, Usha Rani Kandula

**Affiliations:** 1 Department of Psychiatry, College of Health Science and Medicine, Arsi University, Asella, Ethiopia; 2 Department of Nursing, College of Health Science and Medicine, Arsi University, Asella, Ethiopia; UCSI University, MALAYSIA

## Abstract

**Background:**

Medication adherence is the first and main determinant of treatment success. It is defined by world health organization as “the degree to which the person’s behavior corresponds to the agreed recommendations from a health care provider”. Non-adherence is a multi-factorial phenomenon that can result from five major interacting factors. These are health team and health system-related factors; patient-related factors; therapy-related factors; socio-economic factors; and condition-related factors. The prevalence of non-adherence in mental illness was found to be 40% to 60% world wide. In developing countries, the magnitude of poor adherence is expected to increase. So this study aimed to assess medication adherence status and its associated factors among psychiatric patients in Asella Referral and Teaching Hospital in Oromia, Ethiopia.

**Methods:**

An institution-based cross-sectional study was conducted from March 18, 2022 to May 25, 2022, with a total sample of 422 patients. Medication adherence was measured by a modified version of the medication adherence rating scale in the psychiatric setting to determine treatment adherence status, and unstructured questionnaires were assessed by interviewing the patient. Additional data concerning the medication-taking behavior of the patient was collected from caregivers. Bivariate logistic regression was performed to see the association between each explanatory variable and the outcome variable. The odds ratio and 95% confidence interval were used to see the association between treatment adherence and the strength of the link.

**Results:**

A total of 395 study participants were interviewed, making a response rate of 93.6%. The prevalence of treatment adherence was 246(62.3%). Medication adherence show high association with lifetime alcohol use [AOR: 3.18, 95% CI:1.31–7.72] compared to those who had no alcohol use histroy, and perceived stigma [AOR (95% CI: 2.31 (1.01–5.31)] compared with those who had no perceived stigma, where as adherence show low association with having slight or superficial insight about illness [AOR (95% CI: 0.25 (0.12–0.53)] compared to those who reported cured off their illness and belief in medication [AOR: 0.36, 95% CI: 0.16–0.81)] compared to those who didn’t belief in the medication they are taking.

**Conclusion:**

The prevalence of mediation adherence was found to be lower. In this study, factors such as having the slight insight or poor insight about their illness and belief in the medication decreased medication adherence, whereas having an alcohol use history in their lifetime and perceived stigma increased medication adherence. For a better health outcome, awareness creation at an insight level needs to be worked on by psychiatric professionals working on the follow-up psychiatric patients at psychiatry clinic of Assela Referral and Teaching Hospital to enable them to well adhere to their medication.

## Introduction

Medication adherence is the frist and main determinant of treatment success. It is defined by world health organization as “the degree to which the person’s behavior corresponds to the agreed recommendations from a health care provider”. Non-adherence to medicine is a major public health problem that is termed an "invisible epidemics”.

The prevalence of non-adherence in mental illness was found to be 40% to 60% world wide [1]. In developing countries, the magnitude of poor adherence is expected to increase, and its impact will be very high. In Ethiopia, the prevalence of non-adherence was reported as 39.3%–55.5%, and many factors were significantly associated with treatment adherence [3, 6–9]. Non adherence to medication continues to be a significant challenge for patients suffering from mental illnesses, physicians, and the healthcare system [1]. It is well known that many patients have difficulty in following treatment recommendations, especially with mental illness [2]. This is due to some differences from other areas of medicine, such as chronicity with a time of exacerbation and relapse, poor insight into the illness process, patients feeling better and cognitive disturbance, and alternative treatment choices which is the traditional one [3–5]. In particular, in severe mental illnesses like schizophrenia, it is a common and critical issue that needs a solution [2].

Poor adherence is a multi-factorial phenomenon which can result from five major interacting factors; health team and health system related, patient related, therapy related, social /economic and clinical factors [2]. According to some research findings poor adherence in mental illness is associated with poor social support, higher self-stigma, perceived spiritual causation of mental illness and being employed [3, 4]. Of these health system related factors may vary from country to country or even from area to area within a given country where the factors may include lack of health insurance coverage, due to different barrier to high quality health care and having lower socio economic status [6].

Inability to well adhere to a medication regimen may not affect only the patient, it also affect health care effectiveness. Broadly it may result in poor health, higher healthcare costs, relapse of illnesses, increase risk of suicide, increased risk of dependency, presence of inter episodic symptoms, frequent hospital admission, difficulty to return back to previous functionality, increased risk of absents and rebound effect and increase rate of mortality [1].

Because of the multifactorial reasons for non-adherence, also in our country, many risk factors might exist because of the difference in socio-demographic and economic factors, patient-related, health care and health system related, and clinical-related factors. By this time, people even fear going to health facilities due to the COVID-19 pandemic, so it is also the other hindering factor.

Despite the existence of solutions for treatment non-adherence and its determinants, since it is perceived as a minor health issue even if it leads to a variety of undesirable health consequences, there is a scarcity of information on the area of mental illness in our country, particularly in the Arsi Zone, Oromia region to our knowledge of searching. Therefore, we were interested in determining medication adherence status and factors associated with mental illness. The current study will have a significant contribution in that it will help to set strategies to overcome perceptual and practical barriers to adherence. Since it is an indicator of healthcare effectiveness, it can also enhance the flow of patient visits to psychiatry clinics. It involves good patient outcomes and support health policy-making issues.

## Methods and materials

### Study setting

The study was conducted at Asella Referral and Teaching Hospital’s (ARTH) outpatient psychiatric clinic. Asella is a town in Ethiopia’s Oromia regional state, and it is the capital city of Arsi zone. It is 175 kilometers North of Addis Ababa, the country’s capital. ARTH is a well-known government institution in Asella town, and it is a teaching and tertiary level hospital that provides inpatient and outpatient health services for over 3.5 million people in Ethiopia’s North East [7]. More than 60,000 patients got psychiatric service from this hospital. This care is given by 1 psychiatrist, 4 BSC psychiatric nurses, and 2 MSc psychiatry professionals. There is no inpatient psychiatric service in this hospital.

### Study period and design

A hospital-based cross-sectional study was conducted in the psychiatric OPD of ARTH from March 18, 2022 to May 25, 2022.

### Source and study population

The source population were patients who came for follow-up to take medication at Asella Referral and Teaching Hospital. All psychiatric patients who came to take their treatment during the data collection period are included in the study population.

### Eligibility criteria

The study included all patients aged 15 and above who came for follow-up at the psychiatric clinic and whose charts clearly indicated a diagnosis of major mental illness, who were calm and took their treatment on their second visit and on follow-up. On the other hand patients who were not able to communicate because of their illness were excluded.

### Study variables

The treatment adherence status of psychiatric patients, which was assessed by a validated and modified version of the medication adherence rating scale and classified as adherent or non-adherent, was a dependent variable. whereas socio-demographic and economic factors (age, gender, income, occupation, education, religion, ethnicity, area of residence, availability of social support), patient-related factors (smoking, alcohol use, other substance use, patient attitude, belief on medication, fear of stigma, level of satisfaction, and fear of COVID-19 transmission), health-system-related factors (time duration for follow-up, creation of awareness on treatment adherence, availability of medication) and clinical factors such as duration of the illness, insight level, severity of illness, side effect of medication, dosage, frequency per day) were independent variables.

#### Operational definition

- Major mental illness: mental illness with the diagnosis of major depressive disorder, schizophrenia, Bipolar disorders, brief psychotic disorder and schizophreniform
- Medication adherence status: Medication adherence was measured by modified version of medication adherence rating scale with [8] with a total of 8 item where item 1 to 7 have choice of Yes = 1, No = 0 whereas item number 8 have choices A to E, A = 0, B to E = 1. Those who score 8 out of 8 are termed adherents, where a score of less than 8 indicates non-adherence
- Poor adherent- score of less than 6 (0–5)
- Partially adherent–score of 6 to 7
- Adherent– 8 out of 8 score
- Psychiatry patient: patient with major mental illnesses
- Associated factor: factor that lead the patient for non-adherence
- Patients: clients 15 years old and above

### Sample size estimation/determination/

The sample size was calculated using the single population proportion formula. By using the proportion of treatment non-adherence for major mental illness in concurrent cases and for associated factors (since there is no similar study in Ethiopia up to the knowledge of the investigators) of 50%, and 95% confidence level, 5% tolerable margin of error and a possible non-response rate of 10%, the final sample size was 422.

### Sampling procedure

Simple randam sampling technique was used. During the data collection dates, when patients’ charts were brought to the clinic, they were checked for inclusion criteria. The charts were differentiated from new patients, then, by lottery method or randomly, we chose those who would be involved in the study. If a patient is absent while the chart is brought, another patient who fulfills the criteria is added. The patients on follow-up recorded in the record book were the sampling frame of this study.

### Data collection procedure

Data was prepared in English, then it was translated in to local language, Afan Oromo and Amharic and back to English. Data was collected by the preference of patients by either of the two language from respondents. Primary data was collected from individual patients. These were structured questions where the contents were socio-demographic and economic factors; clinical-related factors; health system-related factors; and patient-related factors. In addition to this, four questions concerning unresolved reasons for not properly taking medication at family level and possible solutions they expect were developed to be gathered from care givers. For those patients who came alone, questionnaires designed to be collected from caregivers were collected by phone. Secondary data was gathered from the patient’s chart by using the already prepared checklist that contains 7 items in English. The data was collected by six psychiatric professionals. The principal and coinvestigators provided supervision during the data collection period.

### Data quality assurance

Medication adherence was measured by validated and modified version of the medication adherence rating scale with internal consistency (Cronbach’s alpha) = 0.75 and reliability of 0.83) [8]. Additionally, other questionnaires were prepared after reviewing literature in English. Then it was translated into Amharic and Afan Oromo and back to English to ensure consistency. Data collection took place by Afan Oromo and Amharic languages according to the language fluency of patients. Before starting the data collection, a pre-test of questionnaires was done on 10% of psychiatric patients attending service at Adama General Hospital to prevent cross-contamination of information, and it was performed in order to check the language clarity and consistency of the questionnaire. Corrections to words on the questionnaire that were ambiguous for respondents were made after the pre-test.

There was supervision. The questions’ completeness was checked and corrected before the patients left the mental health clinic. For uniformity and accuracy, the collected data was entered into a computer by utilizing a double data entry procedure.

### Data processing and analysis

The data was cleaned for completeness, coded, entered, and analyzed using the Statistical Package for Social Science (SPSS) version 24.00. The summary of the descriptive analysis was calculated and displayed as frequency and percentage (categorical data), and mean for continuous data. Bivariate analysis was performed to see the association between the independent and the outcome variable. Multi-collinearity was checked to see if there was an association between independent variables and those with a variance inflation factor (VIF) value of 1 to 5 were taken, as they have no correlation, so they were used in the final model. Variables with a p-value of less than 0.25 in the bivariate analysis were candidate variables for multivariable analysis. To identify significant factors in the model, first the variables were filtered by using the backward LR, and then, after some variables were removed, the remaining variables were processed by the enter method. Model fitness was assessed using the Hosmer and Lemeshow test. After adjustment, an independent variable with a p value less than or equal to 0.05 was considered significantly associated with the outcome variable. It was reported using an adjusted odds ratio with a 95% confidence interval.

### Ethical consideration

The Arsi University College of Health and Medical Science Ethical Review Committee granted us ethical approval. All adult participants provided verbal informed consent, and minor participants provided informed consent after receiving a detailed description of the study from their parents/legal guardians. All methods were carried out in accordance with the applicable guidelines and regulations. Patients identified as non-adherent groups were advised to properly take their medication, and family members caring for the patients were given health information.

## Results

### Socio-demographic and economic factors

A total of 395 patients were interviewed, which makes a response rate of 93.6%. Two hundred twenty-nine (58%) of the respondents were females. The minimum age was 15, whereas the maximum age was 78, with a mean age of 33 and a standard deviation of +11.7. A large number of the respondents lie in the age group of 30–39, which accounts for 127 (32.2%). Most of them are Muslim in religion and Oromo in ethnicity; 235 (59.5%) and 322 (81.5%) respectively. Most were from rural areas (56.7%). Two hundred thirty-five (59.5%) of respondents said they had only learned in elementary school, while 43 (10.9%) said they had a diploma or higher. The mean income was 1110.30 Ethiopian Birr (ETB) with a standard deviation of +2065.55ETB. Almost two thirds of the respondents had no well-known income; they account for 293 (74.2%), whereas 3 (0.8%) had the maximum range of income in the group, which lies between 10,055 and 11,305 ETB. More than half of the respondents were married 206 (52.2%). Regarding the presence of an individual to remind them to take their medication, 358 (90.6%) reported that they had an individual who reminded them to take their medication (Table 1).

**Table 1:** Frequency distribution of respondents by socio demographic and economic factors in Asella Referral and Teaching Hospital 2022.

Variables	Categories	Frequency	Percent (%)
Sex	Male	166	42
	Female	229	58
Age	15–19	32	8.1
	20–29	125	31.6
	30–39	127	32.2
	40–49	62	15.7
	50–59	49	12.5
Religion	Christian	160	40.5
	Muslim	235	59.5
Ethnicity	Oromo	322	81.5
	Amhara and other	73	18.5
Residence	Urban	171	43.3
	Rural	224	56.7
Education	Have no formal education	62	15.7
	Grade 1–8	235	59.5
	Grade 9–12	55	13.9
	Diploma and above	43	10.9
Marital status	Single	154	39
	Married	206	52.2
	Other (Divorced and widowed)	35	8.9
Living With	Alone	36	9.1
	With family	315	89.9
	Other (with non-supportive individual)	44	11

### Patient related factors

Among the respondents, 41 (10.4%), 34 (8.6%) and 38 (9.6%) had a history of Khat, tobacco and alcohol use in their lifetimes, respectively. Other substances like cannabis and marijuana were rarely used by 12 (3%) of the study participants. In terms of patients’ belief in the medication they are taking, 354 (89.6%) believed that the medication could cure them. 264 (66.8%) of the respondents have a good understanding of the reason they took the medication. 37 (9.4%) of them reported perceived stigma, while the rest did not. During the time of the CVD-19 pandemic, only 24 (6.1%) reported fear of coming for follow up, whereas 10 (2.5%) had a history of cutting down their medication due to fear of transmission. Even iff the pandemic will continue, 388 people (98.2%) said they would keep taking their medication on follow up. Only 20 (5.1%) were unsatisfied with the service they obtained from the psychiatry OPD (Table 2).

**Table 2:** Frequency distribution of respondents by patient related factors in Asela Referral and Teaching Hospital 2022.

Variables	Categories	Frequency	Present
Life time Chat Use	Yes	41	10.4
	No	354	89.6
Current Chat use	Yes	31	7.8
	No	364	92.2
Life time Alcohol use	Yes	38	9.6
	No	357	90.4
Current Alcohol use	Yes	37	9.4
	No	358	90.6
Life time Tobacco use	Yes	34	8.6
	No	361	91.4
Current Tobacco use	Yes	26	6.6
	No	369	93.4
Other substance (Marijuana and Shisha) use	Yes	12	3
	No	383	97
Belief in medication	Have belief /positive/	354	89.6
	Have no belief/negative/	41	10.4
Reason for taking medication	Good	264	66.8
	Poor	131	33.2
Perceived Stigma	Yes	37	9.4
	No	358	90.6
Having fear of CVD 19 while coming for follow up	Yes	24	6.1
	No	371	93.9
Had history of stopping Follow up due to CVD 19	Yes	10	2.5
	No	385	97.5
Current fear of CVD19 transmission	Yes	26	6.6
	No	369	93.4
Feature plan to Continuing treatment with the pandemic	Yes	388	98.2
	I will stop	7	1.8
Satisfaction	Satisfied	375	94.9
	Unsatisfied	20	5.1

### Health system related factors

Among the study participants, 131 (33.2%) reported that there is a problem with medication accessibility in the hospital pharmacy and 145 (36.7%) of the respondents reported that the cost is expensive. Only 86 (21.8%) reported that they waited more than two hours while they came for their follow up. 263 (66.6%) and 344 (87.1%) were confirmed as they got information from professionals working in the clinic on the side effects of medication and medication adherence (Table 3).

**Table 3:** Frequency distribution of respondents by health system related factors in Asella Referral and Teaching Hospital 2022.

Variables	Categories	Number	Percent
Medication availability problem	Yes	131	33.2
	No	264	66.8
Cost of medication in the hospital pharmacy	Expensive	145	36.7
	Cheap	208	52.7
	Unknown I use insurance	42	10.6
Costly level of the medication in the hospital pharmacy	Extremely expensive	53	13.4
	Medium cost	101	25.6
	Balanced cost	199	50.4
	Unknown I use insurance	42	10.6
Follow up waiting time	Above 2hour	86	21.8
	Below 2hour	309	78.2
Information on side	Yes I got	263	66.6
effect of medication by professionals	No I didn’t got	132	33.4
Information about medication	Yes I got	344	87.1
adherence by professionals	No I didn’t got	51	12.9

### Clinical factors

According to the information obtained from their chart, 255 (64.6%) were diagnosed as cases of schizophrenia and taking atypical antipsychotics, 151 (38.2%) and 122 (30.9%) were on conventional antipsychotics. One hundred sixty-nine (42.8%) of respondents had slight or superficial insight about their illness, and 78 (19.7%) reported being cured of their illness. Concerning severity estimation by the patients, 271 (68.6%) reported that their illness was severe and 49 (12.4%) reported that it is mild. Thirty patients have different reports of side effects currently which was also reported by professional observation. Medical illness comorbidity accounted for 24 (6.1%) of the 27 (6.6%) with high epilepsy comorbidity (Table 4).

**Table 4:** Frequency distribution of respondents by clinical factors in Asella Referral and Teaching Hospital 2022.

Variables	Categories	Frequency	
		Number	Percent
Duration of illness	6–58	159	40.3
	59–111	104	26.3
	112–164	47	11.9
	165–217	44	11.1
	218–480	41	10.4
Insight	Slight (superficial) insight	169	42.8
	Poor /no insight	148	37.5
	Say cured	78	19.7
Severity Rank by respondents	Severe	271	68.6
	Moderate	75	19
	Mild	49	12.4

Diagnosis of the respondent	Schizophrenia	255	64.6
	Other psychotic disorder	34	8.6
	MDD and other DD	59	14.9
	Bipolar disorder	25	6.3
	Anxiety disorders	22	5.6
Medication the respondent is taking	Atypical antipsychotics	151	38.2
	Conventional antipsychotics	122	30.9
	Antidepressant	36	9.1
	Combination of antipsychotics and antidepressant	69	17.5
	Combination of antipsychotics and Mood stabilizer	17	4.3
Medication side effect	Yes	60	15.2
	No	335	84.8
Medication frequency	Once per day	345	87.3
	Twice per day	41	10.4
	Other option (injectable, once per month)	9	2.3
Type of side effect	Tremor (hand and body)	7	1.8
	Weight gain	9	2.3
	Other (dry mouth, drowsiness and sedation)	4	1
	Drawling of saliva, muscular rigidity	1	0.3
	Other (Malignant)	9	2.3
	No side effect yet	365	92.4
Type of Comorbid illness	Diabetes Miletus	5	1.3
	Hypertension	2	0.5
	Epilepsy and other neurologic disorder	12	3
	Other (Liver, kidney and heart problem)	5	1.3
	No comorbid illness	371	93.9

MDD = Major depressive Disorder, DD Depressive disorder

### Information taken from caregivers to obtain additional data

All respondents were interviewed face-to-face and by phone to add additional information that may be associated with the medication-taking behavior of the respondents. Accordingly, among 395 close family members, 132 (33.4%) reported that the most common reason for not properly taking the medication was a lack of insight and hesitation as a result. For the question asked the difficult and unsolved issue by them not to properly take medication, 130 (32.9%) of care givers reported lack of insight and hesitation as a result was found the difficult and still unsolved problem, followed by care givers’ report that there is no difficult problem that accounts for 109 (27.6%). For the possible solution for proper medication taking behavior, 130 (32.9%) of caregivers reasoned that free and sufficient medication accessibility would be a possible solution, followed by strong family support at 84 (21.3%) (Table 5).

**Table 5:** Frequency distribution of respondents by their caregivers stated factors at Asella Referral and Teaching Hospital 2022.

Variable	Categories	Frequency	
		Number	Percent
The most critical problem for not taking medication properly	financial problem	46	11.6
	Distance, lack of transportation	10	2.5
	Hopelessness	6	1.5
	Forgetting	59	14.9
	lack of insight, including hesitate to take medication	132	33.4
	lack of understanding about treatment adherence	9	2.3
	personal issues like poor social support and substance use	12	3
	religion (like using holly water)	3	0.8
	lack of cure	3	0.8
	belief cured	4	1
	because of side effect	10	2.5
	No reason	101	25.6
Difficult and unsolved problems	Financial problem	51	12.9
	Distance, lack of transportation	14	3.5
	Belief cured	7	1.8
	Personal issues (poor social support, substance use and forgetting)	67	17
	lack of insight, hesitate	130	32.9
	Lack of understanding	9	2.3
	Religion	1	0.3
	Lack of cure	2	0.5
	No difficult reason	109	27.6
	Because of side effect	5	1.3
Possible solutions set by givers	Giving health information	159	40.3
	Free and enough medication	130	32.9
	Shortening waiting time	3	0.8
	I do no	11	2.8
	family support	84	21.3
	deep examination like test with machine	3	0.8
	Follow up should be long enough	5	1.3

### Prevalence of medication adherence status

**Fig 1:**
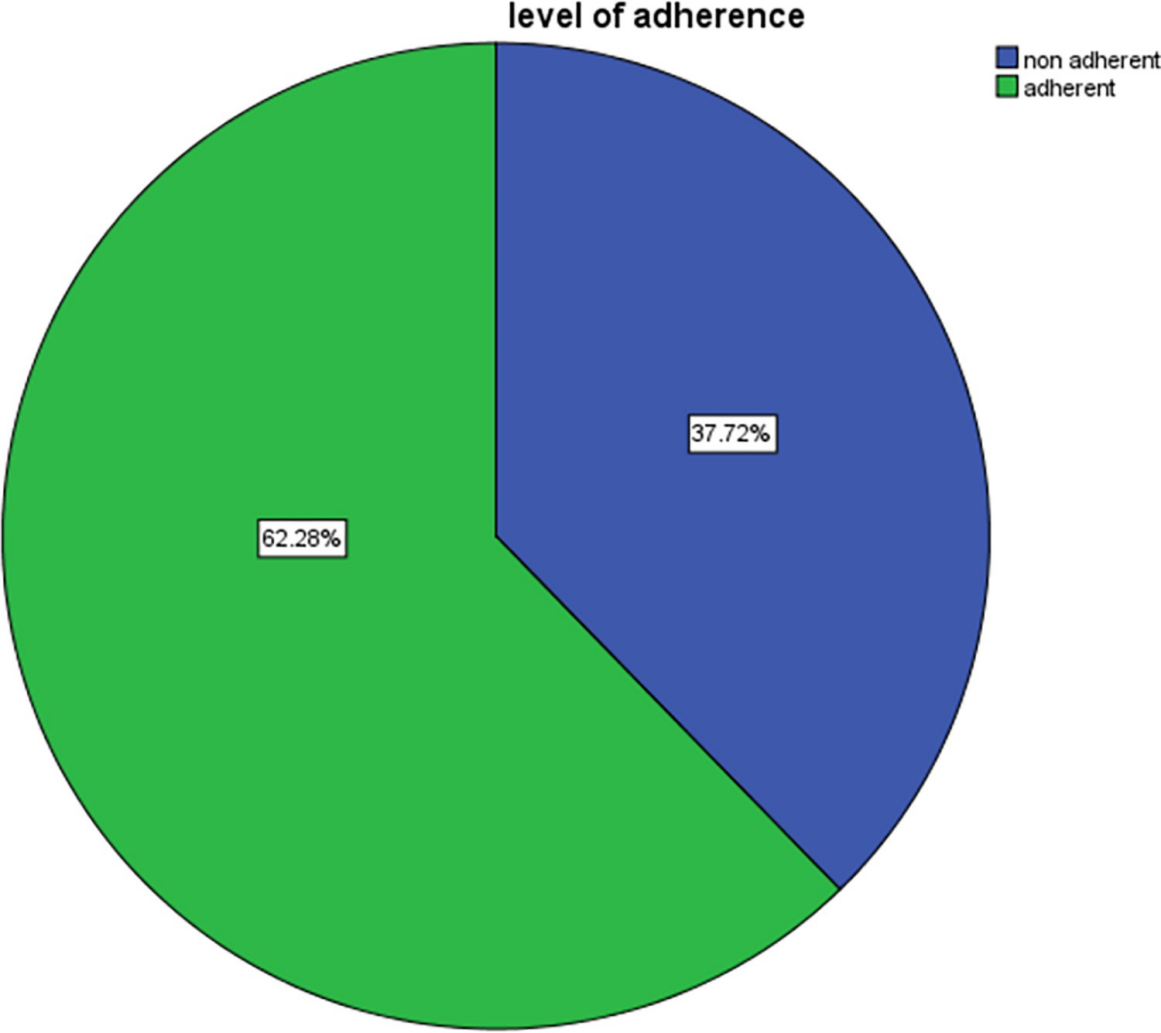
Prevalence of medication adherence status of respondents in Assela Referral and Teaching Hospital.

Fig 1 indicates the prevalence of respondents’ medication adherence status at Assela Referral and Teaching Hospital in Ethiopia. Among the respondents, 149 (37.7%) were non-adherent and 246 (62.3%) of them were adherent to medication.

### Factors associated with treatment adherence

Lifetime alcohol use, belief in medication, perceived stigma, and illness insight were among the variables that showed a significant association with treatment adherence. The odds of medication adherence among respondents who had a lifetime alcohol use history [AOR (95%CI); 3.18 (1.31–7.72)] were 3 times higher than their counterparts. Individuals who believed in their medication were 65% less likely to be adherent than those who did not believe in their medication [AOR (95%CI); 0.36 (0.16–0.81)]. Respondents who had perceived stigma had 2 times higher chance of adherence to their medication compared to those who had no perceived stigma [AOR (95%CI); 2.31 (1.01–5.31)]. Individuals who had slighht insight compared to those who reported currently getting a cure from their illness were less adherent [AOR (95%CI); 0.25 (0.12–0.53)]. The educational status and medication availability problems in the hospitals’ pharmacy were found insignificant in the final model. (Table 6).

**Table 6:** Multivariate logistic regression model for selected factors with treatment adherence status of respondents at Asella Referral and Teaching Hospital 2022.

Variables	Categories	level of adherence		COR (95%CI)	p value	AOR (95%CI)	P Value
		Adherent	Non adherent				
Education	No formal education	29	33	0.47 (0.21–1.04)*	0.06	0.58 (0.24–1.41)	0.23
	grade 1–8	157	78	.82 (1.07–0.54)	0.82	1.27 (0.59–2.72)	0.52
	grade 9–12	32	23	0.75 (0.32–1.7)	0.48	0.97 (0.39–2.39)	0.95
	diploma and above	28	15	1		1	
Life time Alcohol use	Yes	31	7	2.92 (1.25–6.82)*	0.01	3.18 (1.31–7.72)*	0.01
	No	215	142	1		1	
Belief in medication	Yes	213	141	0.36 (0.16–0.81)*	0.01	0.35 (0.15–0.83)*	0.01
	No	33	8	1		1	
perceived stigma	Yes	28	9	1.99 (0.91–4.36)	0.08*	2.31 (1.01–5.31)*	0.04
	No	218	140	1		1	
Medication availability problem	Yes	90	41	1.52 (0.97–2.36)	0.06*	1.55 (0.95–2.50)	0.07
	No	156	108	1		1	
Insight about illness	Slight insight	108	61	0.29 (0.14–0.59)*	0.0011	0.25 (0.12–0.53)*	0.00
	Poor/No insight	71	77	0.15 (0.07–0.3)	0.001	0.12 (0.06–0.26)*	0.00
	Say cured	67	11	1			

*- significantly associated

AOR- Adjusted Odds Ratio

Adjusted factors- life time alcohol use, belief in medication, perceived stigma and insight about illness

## Discussion

The aim of this study was to assess the prevalence of treatment adherence and its associated factors. The prevalence of treatment adherence was found to be 62.3%. This prevalence is nearly similar to a study conducted in Jimma, Nigeria with a prevalence of 60.5% and 55.7% respectively [3, 9]. Where as it was high compared with studies conducted in Bahir Dar at different years of study [10–12] and systematic review and Meta-analysis summary prevalence [11]. The possible reasons for the difference can be explained by differences in client background, time of the study and the tool used for assessing adherence to medication. There are different factors for treatment adherence according to different findings because of variation in different aspects across the world, including setting, clients’ background, tools used, and other factors [11, 13]. In the current study, factors significantly associated with treatment adherence were found to be: life-time alcohol use, perceived stigma, belief about medication, and insight about illness. In this study, lifetime alcohol use history had 3 times the odds of adherence compared to those who had no history of using alcohol in their lifetime. It contradicts a systematic review of the literature, which found substance abuse to be a significant factor in medication non-adherence among patients with various major mental illnesses [4, 14] and excessive alcohol use is related to a lower medication adherence rate [15]. The possible reason for the difference might be due to respondents’ change in attitude toward medication taking as a result of getting help and advice from psychiatric professionals to stack on their medication. Those who had perceived stigma were 2 times more adherent compared to those who had no perceived stigma. This can be explained by the respondents’ inner motivation to be cured of their illness so that people cannot stigmatize them. Belief about medication was the other factor which showed a significant association in which clients who believed in their medication were less adherent compared to those who had no belief in their medication. This can be due to an arbitrary answer by respondents to a closed-ended question. This is contrary to the study finding in New York, which states that higher motivation for mental health treatment and recovery support was related to greater adherence [15, 16]. The difference might be due to fear of responding "I cannot believe in the medication you are giving to me." to the clinician. Some of the respondents may say yes only to prevent further questions. Concerning insight, those respondents who had slight insight and those who had no insight were less adherent to their medication than those who reported they got more cure from their illness. This is supported by studies’ findings which found that poor insight is one of the significant factors in medication non-adherence [4, 5, 9, 10, 14]. It is not consistent with the findings of study which found patients feeling better [4] and expectation of cure rather than continuing treatment leads for treatment non adherence [17].

In this study, additional data was gathered from family members who were caring for the patient to search for possible associated factors for medication non-adherence. This is due to the multifaceted factors that caused patients to deviate from their medication. So, unless care givers are included, it is impossible to solve the problem of non-adherence, which is recommended by some researchers [11, 15, 18]. Therefore, this finding part was supportive of the clients’ data. They stated that the most critical problem for not taking medication properly was a lack of insight, which accounted for 33% of the total. Still, this is the problem that most family members did not have a solution for (33%). While family members asked for possible solutions for their significant other to adhere to their medication, (40%) reported giving health information for the patient followed by free and enough medication from the hospital (33%) might help to solve the current non-adherence issue, according to the caregiver’s opinion. Generally, it is impossible to conclude that adherence can be improved without the involvement of patients and significant others in their family members [18].

### Limitation

Because this study is cross-sectional type, it cannot determine the exact cause of treatment nonadherence and it cannot indicate non adherence effects on the patients. Secondary data were extracted from patients’ charts but not assessed from the patient. Some people were simply saying yes for some of the close ended question.

### Strength

Data were collected by professionals by using local language, translation of questions were done by concerned language department professionals and health information was given for non adherent patients after knowing the result.

## Conclusion

The prevalence of medication adherence among people with major mental illness was not satisfactory. Factors like alcohol use, belief in medication, having perceived stigma and insight were found to be associated with medication adherence status. Alcohol use history and perceived stigma increase medication adherence, whereas having superficial insight, poor insight, and belief in medication decrease medication adherence.

## Recommendation

Improving the medication adherence of people with major mental illness in the study area and tackling those associated factors will have a better contribution to the good outcome of patients. As a result, health care facilities, professionals, patients, and family members need to collaborate to address these problems. Awareness creation at an insight level needs to be worked on by psychiatry professionals working on follow-up of patients at ARTH psychiatry clinic to enable patients to well adhere to their medication. Deep understanding of adherence and true belief in their medication will help to increase their adherence level. Discussing patient-specific factors with the patient and significant other family members will further address the problem of adherence need to be addressed by psychiatry professionals working in this clinic.

## Abbreviations

ARTH: Assela Referral and Teaching Hospital
ART: Anti Retro Viral Therapy
COVD: Corona Virus Disease
ETB: Ethiopian Birr
MMI: Major Mental Illness
OPD: Outpatient Department
SPSS: Software Package for Social Science
TB: Tuberculosis
VIF: Variance inflation factor
WHO: World Health Organization
